# Toxoplasmosis seroprevalence in rheumatoid arthritis patients: A systematic review and meta-analysis

**DOI:** 10.1371/journal.pntd.0006545

**Published:** 2018-06-05

**Authors:** Zahra Hosseininejad, Mehdi Sharif, Shahabeddin Sarvi, Afsaneh Amouei, Seyed Abdollah Hosseini, Tooran Nayeri Chegeni, Davood Anvari, Reza Saberi, Shaban Gohardehi, Azadeh Mizani, Mitra Sadeghi, Ahmad Daryani

**Affiliations:** 1 Toxoplasmosis Research Center, Mazandaran University of Medical Sciences, Sari, Iran; 2 Department of Parasitology, School of Medicine, Mazandaran University of Medical Sciences, Sari, Iran; 3 Student Research Committee, Mazandaran University of Medical Sciences, Sari, Mazandaran, Iran; University of Iowa, UNITED STATES

## Abstract

**Background:**

Toxoplasmosis is a cosmopolitan infection caused by an intracellular obligatory protozoan, *Toxoplasma gondii*. Infection to this parasite in immunocompetent patients is usually asymptomatic, but today it is believed that the infection can be a risk factor for a variety of diseases, including rheumatoid arthritis (RA). RA is an autoimmune disease and the most common type of inflammatory arthritis that is a major cause of disability. The aim of this systematic review and meta-analysis was to address the association between RA and toxoplasmosis in light of the available research.

**Methods:**

Based on the keywords, a systematic search of eight databases was conducted to retrieve the relevant English-language articles. Then, the studies were screened based on the inclusion and exclusion criteria. The random effect model was used to calculate the odds ratio (OR) using forest plot with 95% confidence interval (CI).

**Results:**

Overall, 4168 Individual, extracted from 9 articles were included for systematic review evaluation, with 1369 RA patients (46% positive toxoplasmosis) and 2799 individuals as controls (21% positive toxoplasmosis). Then, eight articles (10 datasets) were used for meta-analysis (1244 rheumatoid arthritis patients and 2799 controls). By random effect model, the combined OR was 3.30 (95% CI: 2.05 to 5.30) with P < 0.0001.

**Conclusion:**

Although toxoplasmosis could be considered as a potential risk factor for rheumatoid arthritis, more and better quality studies are needed to determine the effect of *T. gondii* infection on induction or exacerbation of RA. Our study was registered at the International Prospective Register of Systematic Reviews (PROSPERO; code: CRD42017069384).

## Author summary

*Toxoplasma gondii* is an intracellular obligatory protozoan, which causes toxoplasmosis. *T*. *gondii* infection in immunocompetent individuals is mostly asymptomatic, but it may be reactivated as a result of immune disorders inducing serious complications. Rheumatoid arthritis (RA), as a complex autoimmune disease, is a major cause of significant and progressive disability, articular complications, and premature death. Studies confirmed an interaction between infections and environmental factors as the potential risk or protective factors determining the development of autoimmune diseases. In this study, we investigated the association between toxoplasmosis and RA.

## Introduction

Toxoplasmosis is a parasitic disease with worldwide distribution caused by obligate intracellular coccidian protozoan *Toxoplasma gondii* (*T*. *gondii*) [1]. It is estimated that one-third of the world’s population are infected with this parasite in both developed and developing countries [2, 3]. Humans can be infected with the parasite through different routes, including consumption of raw or undercooked meat containing tissue cysts of the parasite, ingestion of sporulated oocysts from contaminated water and food, and vertical transmission during pregnancy through the placenta to the fetus [4].

*T*. *gondii* remains in the infected host tissues perpetually [5]. Most immunocompetent individuals, if infected with this parasite, are asymptomatic or show minor symptoms [6]. The most common symptom of toxoplasmosis in humans is lymphadenopathy that may be associated with fever, sore throat, muscle pain, fatigue, and headache [4]. In congenitally infected and immunocompromised patients, this disease is more likely to bring about severe complications [7]. Myocarditis and polymyositis have been reported in immunocompetent individuals with acute toxoplasmosis [8]. Furthermore, toxoplasmosis may cause polyarthritis in the hand and knee joints [9]. Polytenosynovitis (inflammation of a tendon sheath) caused by *T*. *gondii* has also been reported [10].

Rheumatoid arthritis (RA) is a common autoimmune disease, which is a major cause of inflammation of the joints and the principal cause of disability that affects 0.5–1% of the population [11, 12]. The disease presents with swollen joints, production of autoantibodies (rheumatoid factor), and systemic effects [13].

In recent years, the role of infectious agents, especially bacteria and viruses, has been identified in the pathogenesis of autoimmune diseases, while the role of parasitic infections due to their vague effects on host immunity has not been well-investigated. Experimental evidence may support the protective effect of specific parasitic infections in the susceptibility to autoimmunity [14]. Some geoepidemiological studies showed that host genetic susceptibility interacts with lifestyle and environmental factors, such as socioeconomic status, dietary habits, environmental pollutants, and ultraviolet radiation exposure; further, infections increase the risk of developing autoimmunity [15]. On the other hand, infectious diseases may contribute to the development of autoimmune diseases through molecular mimicry and epitope spreading [16]. Therefore, the aim of this systematic review and meta-analysis was to provide an updated review of data about the relationship between toxoplasmosis and RA.

## Methods

### Search strategy

This study was performed according to the Preferred Reporting Items for Systematic Reviews and Meta-Analysis (PRISMA) and its checklist [17]. Individuals with RA, along with a control group, were surveyed. To begin, we searched scientific databases for all the articles on the association between toxoplasmosis and RA published up to the first of January 2018. These keywords were used alone or in combination: “*Toxoplasma gondii*”, “toxoplasmosis”, “seroprevalence”, “prevalence”, “rheumatoid arthritis”, “rheumatoid factor”, “meta-analysis”, and “systematic review”.

A literature review was carried out using English databases including “PubMed”, “Google Scholar”, “Science Direct”, “Scopus”, “Web of Science”, “EMBASE”, “CINAHL”, and “ProQuest”. The systematic search of articles was conducted from March 4 to December 31, 2017 by two researchers independently. Also, for completing the checklist, we investigated all the references lists of the selected articles manually. In this study, only English-language articles were analyzed; furthermore, unpublished studies were not evaluated.

### Study selection

After completing the search, the selected articles were reviewed by the two researchers independently. All the duplicate and irrelevant studies were excluded after reviewing the title, abstract, and full text of the articles. Moreover, to prevent reprint bias, the results of the articles were carefully investigated and duplicates were omitted.

### Quality assessment

In order to assess the quality of reporting of the studies, standard Strengthening the Reporting of Observational Studies in Epidemiology checklist (STROBE) was used [18]. S1 Checklist represents the quality score of different eligible studies. This checklist included items assessing the study methodology, study type, study population, sample size, sample collection methods, statistical tests, and presentations. In our study, articles were evaluated based on STROBE assessment (low quality: less than 16.5, moderate quality: 16.6–25.5, and high quality: 25.6–34). The articles we entered in our meta-analysis had acceptable quality.

### Inclusion and exclusion criteria

Abstracts and full texts were assessed independently by the two researchers using a piloted form. The final decisions about the eligibility or exclusion of studies were made separately. Disagreements were resolved with provision for arbitration from a third reviewer. Following the removal of duplicate entries, articles were evaluated according to the following criteria: (1) cohort or case-control studies about the relationship between toxoplasmosis as an exposure and rheumatoid arthritis as a disease, (2) the studies conducted only on humans, (3) the presence of case and control groups, (4) the studies where toxoplasmosis was diagnosed by detecting IgG and**/**or IgM antibodies against *T*. *gondii* in individuals with definitive diagnosis of RA, and (5) the studies providing details on the seroprevalence rate of toxoplasmosis and RA.

The exclusion criteria comprised: (1) studies that were only descriptive, (2) studies that only presented the final result and did not provide the raw data, (3) articles that were not available in English language, and (4) the studies conducted on animals.

### Data extraction

Articles were carefully studied and the following data were extracted: first author, year of publication, the number of patients and controls, the number and percentage of the positive and negative cases of serum IgG and IgM in patients and controls, as well as information about age and gender and laboratory results. In studies where two different populations were studied, data were extracted separately.

### Statistical analysis

The meta-analysis was executed with the Stats Direct statistical software (http://statsdirect.com). For displaying the heterogeneity between studies, χ^2^-based Cochrane test (Q) and I^2^ index were applied [19]. Due to significant heterogeneity between the studies, a random effect model was used to combine the results of the studies. Forest plot was used to indicate the prevalence of toxoplasmosis in each study and to determine pooled estimate prevalence in the studies. Odds ratios (ORs) and 95% confidence intervals (CI) were used for estimating the risk of *T*. *gondii* infection (the significance of P<0.05). OR > 1 indicates the positive effect of *Toxoplasma* on RA and an OR < 1 shows that toxoplasmosis has a protective effect against RA. Publication bias was examined by funnel plots and the statistical significance was assessed by the Egger test [20]. Also, it was performed a sensitivity analysis to identify probably effect of each article on the overall results by excluding them using Stata version 14 (Stata Corp, College Station, TX, USA).

The study protocol (CRD42017069384) was registered on the website of the International Prospective Register of Systematic Reviews (PROSPERO) [21].

## Results

**Fig 1:**
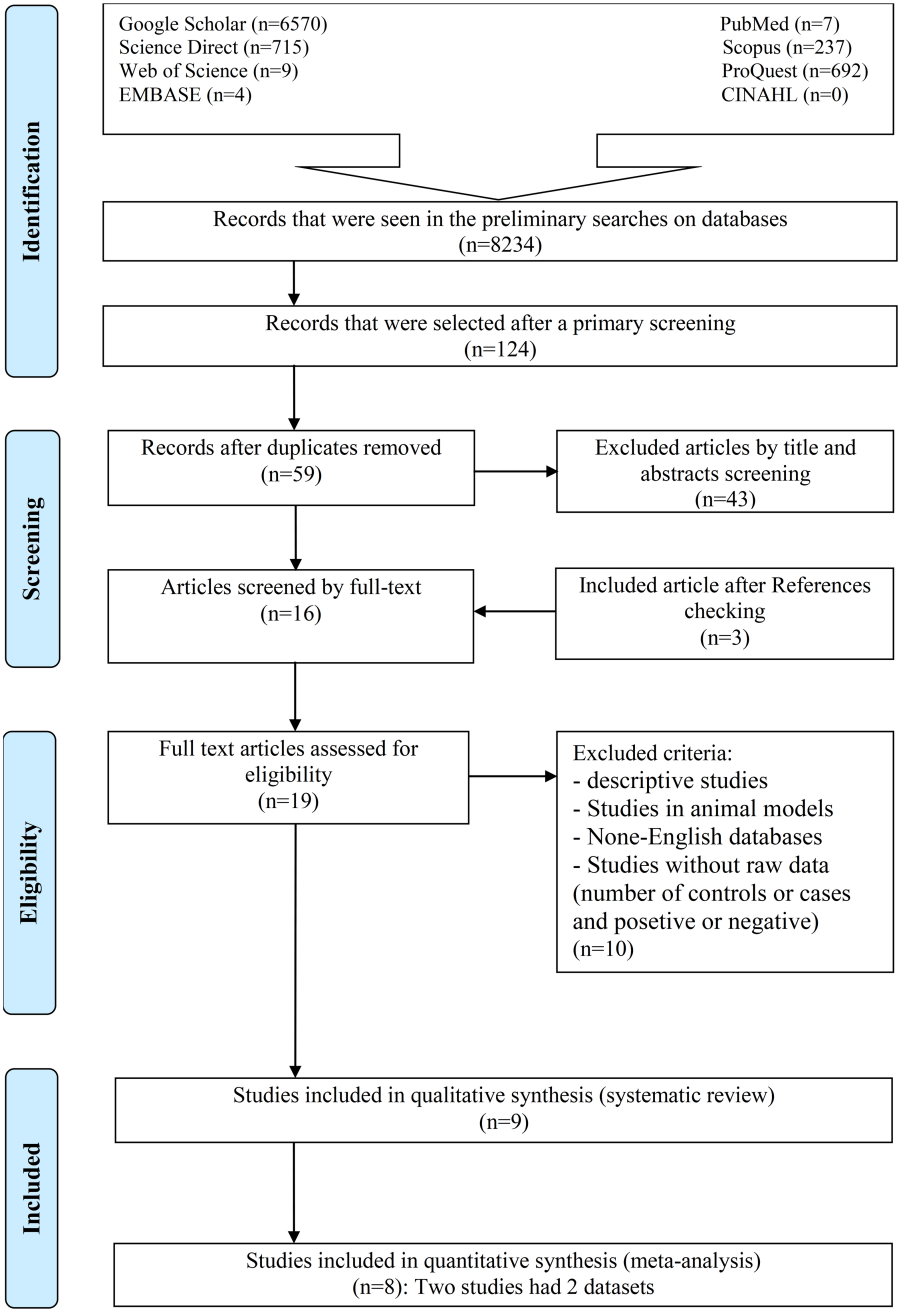
Flow diagram of the study design process.

Our preliminary search of eight databases yielded 8234 papers. After a primary screening of the titles of the articles based on keywords, 124 studies were extracted. Sixty-five articles were also excluded from the study due to duplication. In the next step, by screening the abstracts of the articles and based on the inclusion/exclusion criteria, 43 other articles were excluded. After reading the full text of the articles, 10 other papers were omitted, and three studies were added to the collection after reviewing the references. After the final review of the articles, nine eligible studies [14, 16, 22–28] were identified for systematic review. Another study was excluded due to the absence of a healthy control group [16]. Finally, eight of these nine articles [14, 22–28] were entered into the meta-analysis with respect to the inclusion/exclusion criteria (Fig 1). The studied articles were published between 2007 and 2017. We identified 11 datasets from the nine articles that met the inclusion criteria, eight of which were case-control, two cross-sectional, and one were cohort studies (Table 1). The surveys were conducted in Latin America [14], Europe [14, 16], Egypt [24, 25], Iraq [22, 23, 27], Czech and Slovak [26], and China [28].

**Table 1:** Baseline characteristics of the included studies in the systematic review and meta-analysis of the relationship between *T*. *gondii* infection and RA patients.

No	First author	Publication year	Place of study	Type of study	Method	Test	Results	Age	Sex
**1**	Shapira Y [14]	2012	Europe	Case control	BioPlex 2200 system	IgG IgM	Significant	----	----
**2**	Shapira Y [14]	2012	Latin America	Case control	BioPlex 2200 system	IgG IgM	Not significant	----	----
**3**	Fischer S [16]	2013	Europe	Cross sectional	Chemi luminescence	IgG	Significant	P: 40–74 C:--	----
**4**	Kuba RH [27]	2014	Iraq (Treated patients)	Case control	ELISA	IgG IgM	Significant	P: 20–80 C:--	----
**5**	Kuba RH [27]	2014	Iraq (Untreated patients)	Case control	ELISA	IgG IgM	Significant	20–80 C:--	----
**6**	Al kalaby RF [22]	2016	Iraq	Case control	ELISA	IgG IgM	Significant	P: 16–50 C: 16–50	F
**7**	El-Sayed NM [25]	2016	Egypt	Case control	EIA	IgG IgM	Significant	P: 30–58 C: 29–57	P: (F:70, M:30) C: (F:34, M:16)
**8**	Flegr J [26]	2016	Czech and Slovak	cohort	ELISA CFT	IgG IgM	Significant	----	P: (F:10, M:3) C: (F:935, M:372)
**9**	El- Henawy AA [24]	2017	Egypt	Cross sectional	ELISA	IgG IgM	Significant	P: <60 C: <60	P: (F:29, M:31) C: (F:28, M:32)
**10**	Tian A-L [28]	2017	China	Case control	ELISA	IgG IgM	Significant	----	P: (—) C: (F:454, M:366)
**11**	Al- Oqaily MA [23]	2017	Iraq	Case control	ELISA	IgG IgM	Significant	P: 13–68 C: 13–68	----

Age is in years, ELISA: enzyme-linked immunosorbent assay, EIA: enzyme immunoassay, CFT: complement fixation test, IgG: Immunoglobulin G, IgM: Immunoglobulin M, P: Patient, C: Control, F: Female, M: Male

Our meta-analysis was performed among 4168 people including 1369 RA patients and 2799 controls. In all the studies, blood samples were collected from patients and controls. To identify anti-*Toxoplasma* antibodies (IgG and IgM) in those studies, ELISA [22–24, 26–28], CFT [26], EIA [25], chemiluminescence [16], and BioPlex 2200 system [14] were used (Table 1).

Except for Fischer et al. [16], who only evaluated IgG, other authors surveyed both IgG and IgM antibodies. However, only three studies had reported a titer of antibodies [23–25], and others had described the percentage of positive antibodies in patients and controls. Finally, all the studies analyzed the relationship between toxoplasmosis and RA with respect to the percentage of seropositive and seronegative individuals (patients and controls).

**Fig 2:**
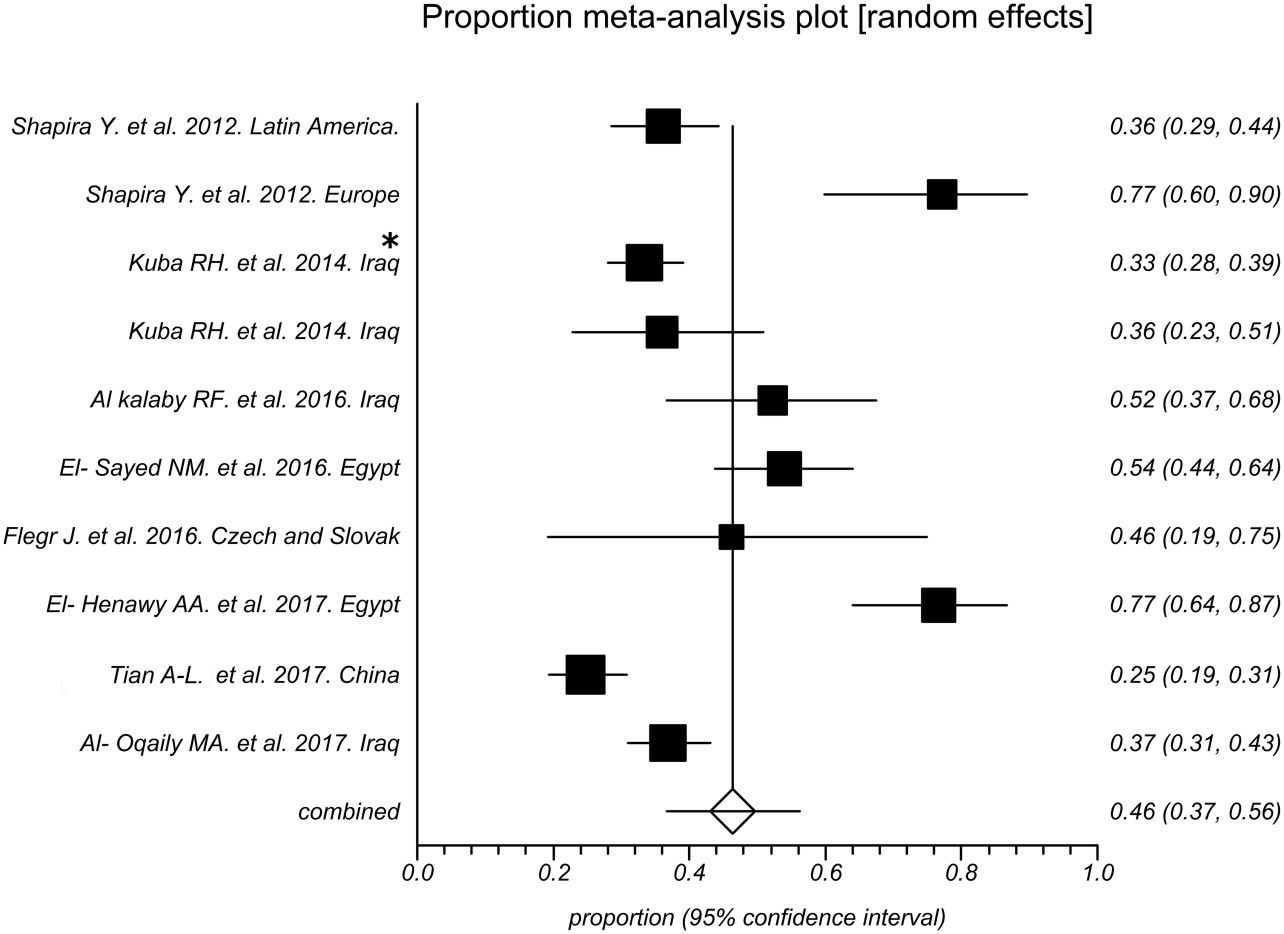
Forest plot of seroprevalence rates of toxoplasmosis in rheumatoid arthritis patients. * Patients under treatment.

**Fig 3:**
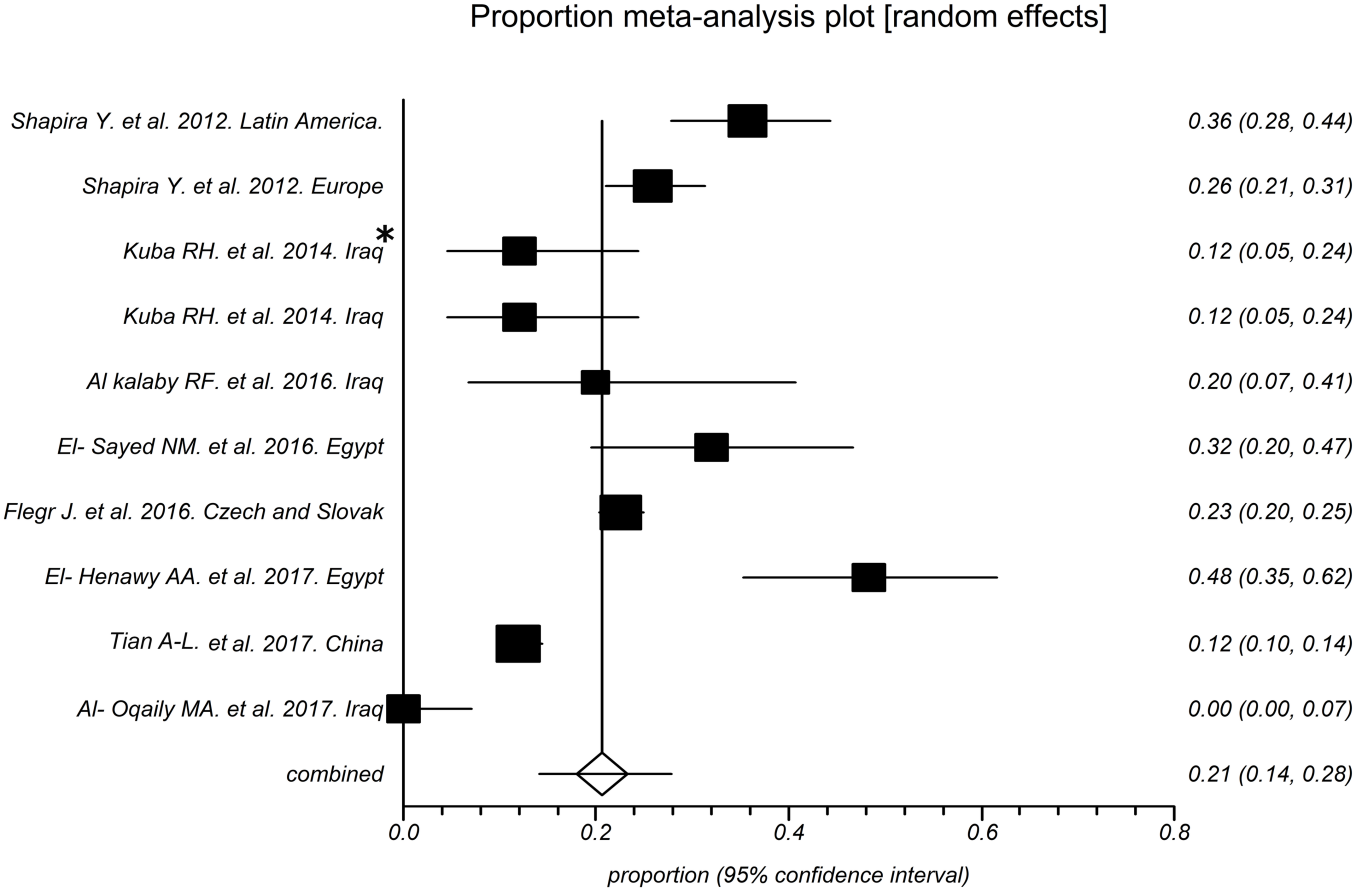
Forest plot of seroprevalence rates of toxoplasmosis in controls groups. * Patients under treatment.

As shown in Fig 2, the prevalence of toxoplasmosis in RA patients in these studies varied from 25% to 77% with an overall seroprevalence of 46% (95% CI [37; 56]). However, the total prevalence of this disease in the control subjects entered in these studies was 21% (95% CI [14; 28]), which varied from 0% to 48% in various studies (Fig 3).

**Fig 4:**
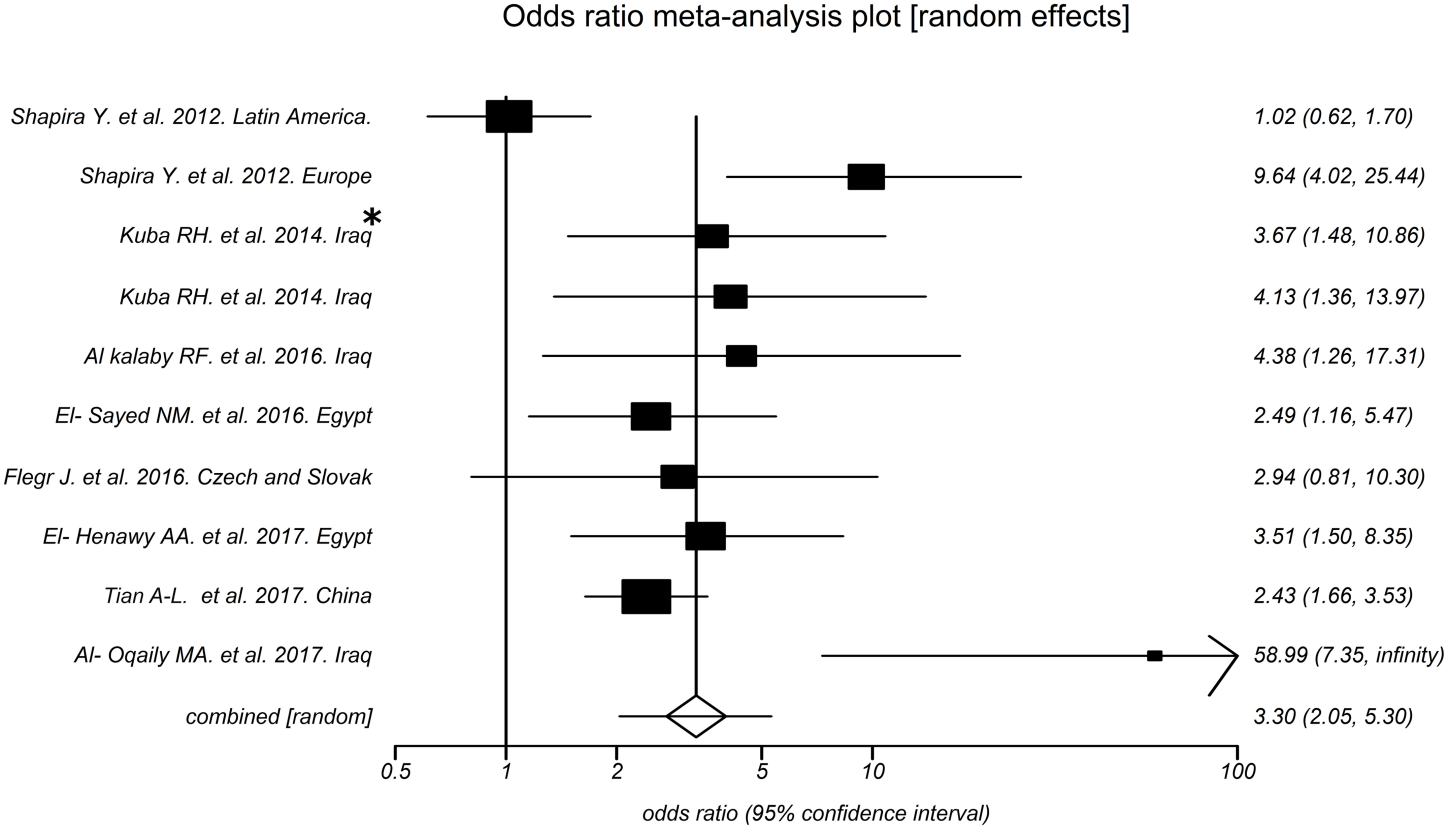
Forest plot of odds ratios for correlation between toxoplasmosis and rheumatoid arthritis. * Patients under treatment.

According to Fig 4, the odds of toxoplasmosis in RA patients are 3.30 times compared to that of controls with 95% CI: 2.05 to 5.30 and P < 0.0001. Nonetheless, the heterogeneity analysis of the effect size of arthritis (Q = 32.77, P = 0.0001, I^2^ = 72.5%) showed a relatively high heterogeneity in our meta-analysis.

**Fig 5:**
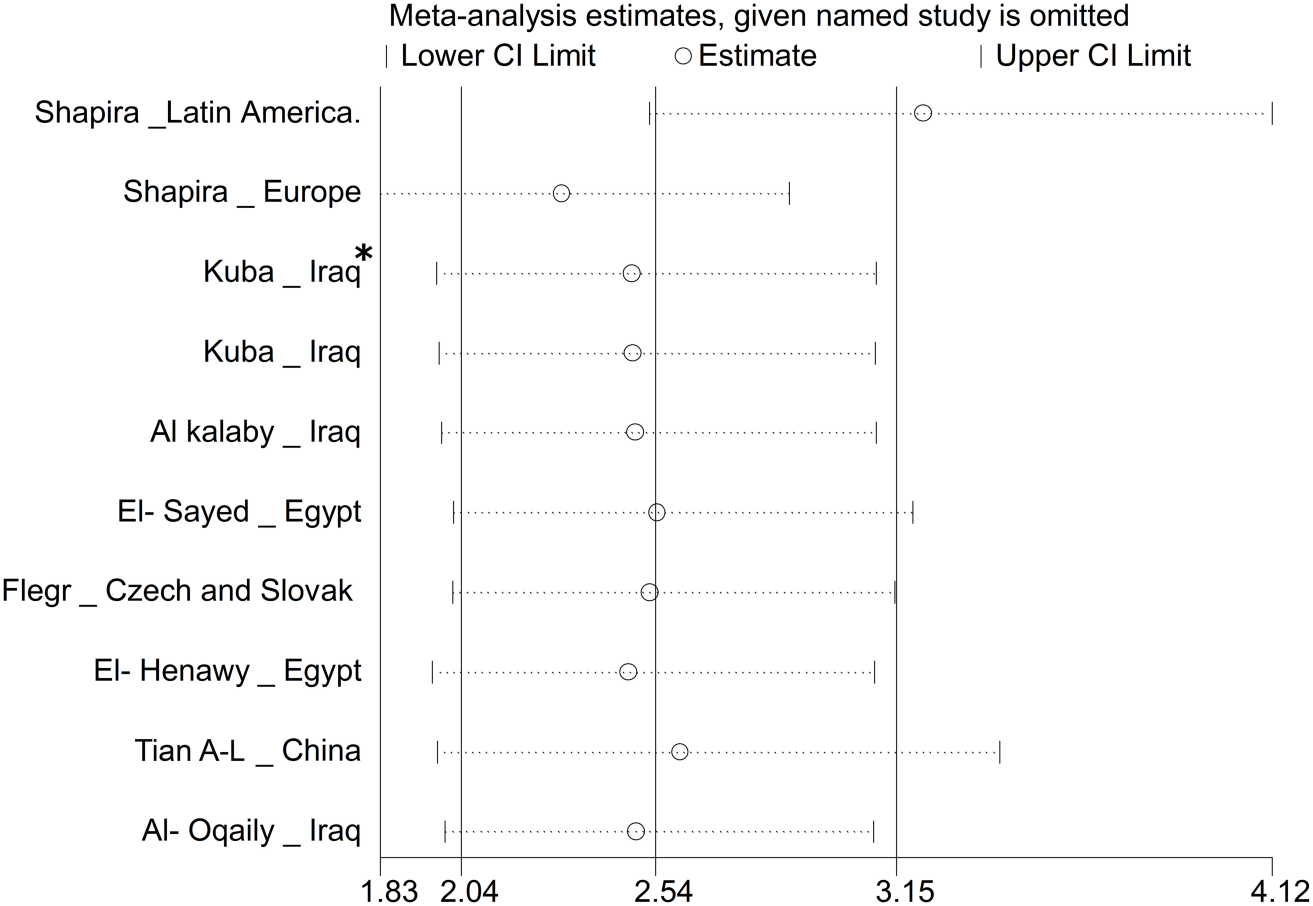
Sensitivity analysis for assessing the effect of each primary study on the total estimates. * Patients under treatment.

Begg and Egger tests were used to evaluate publication bias. Negligible publication bias was observed using both Begg test (P = 0.0286) and Egger test (P = 0.0446) in the included studies. The results of sensitivity analysis showed that the impact of each study on meta-analysis was not significant on overall estimates (Fig 5).

## Discussion

*Toxoplasma gondii* is an important opportunistic parasite infecting one-third of the world’s human population and it is considered a silent threat [29]. Though a clear relationship between toxoplasmosis and autoimmune diseases, including RA, has not yet been well documented, a higher prevalence of anti-*T. gondii* antibodies was reported in patients with rheumatoid arthritis [16]. Thus, we designed this systematic review and meta-analysis to explore the possible association between *Toxoplasma* infection and RA, an autoimmune disease causing pain and disability [30].

Although few studies were included in our meta-analysis, our findings showed that the prevalence of toxoplasmosis in the control group was 21%, which is almost in agreement with the results obtained by Dubey and Beattie [31]. This seroprevalence was significantly different from the prevalence in RA patients (46%).

According to Table 2, the lowest OR was reported in Latin America and the highest OR in Europe. The difference in ORs can be attributed to the significant difference in host response and virulence of parasitic strains [32]. In addition, we found high heterogeneity in the relationship between RA and *T*. *gondii* infection in this systematic review. The high heterogeneity index is suggestive of potential variation, which could be due to difference in genetic potential of humans, which is affected by lifestyle and environmental factors such as dietary habits, environmental pollution, exposure to ultraviolet radiation, various types of infections, and socioeconomic status [33].

**Table 2:** Data extracted from the included studies in the meta-analysis for an association between toxoplasmosis and RA.

No	Reference	N	Case: RA+ (n)	Control: RA- (n)	RA+ & T+ (n, %)	RA- & T+ (n, %)	OR (95% CI)	P-value
**1**	Shapira Y [14] (Latin America)	292	152	140	55 (36.18%)	50 (35.71%)	1.02 (0.62–1.70)	NS
**2**	Shapira Y [14] (Europe)	332	35	297	27 (77.14%)	77 (25.93%)	9.64 (4.02–25.44)	< 0.0001
**3**	Kuba RH [27] (Treated)	344	294	50	98 (33.33%)	6 (12%)	3.67 (1.48–10.86)	< 0.05
**4**	Kuba RH [27] (Untreated)	100	50	50	18 (36%)	6 (12%)	4.13 (1.36–13.97)	< 0.05
**5**	Al kalaby RF [22]	69	44	25	23 (52.27%)	5 (20%)	4.38 (1.26–17.31)	0.01
**6**	El-Sayed NM [25]	150	100	50	54 (54%)	16 (32%)	2.49 (1.16–5.47)	S
**7**	Flegr J [26]	1320	301	1019	6 (46.15%)	295 (22.57%)	2.94 (0.81–10.30)	0.012
**8**	El- Henawy AA [24]	120	60	60	46 (76.67%)	29 (48.3%)	3.51 (1.50–8.35)	< 0.001
**9**	Tian A-L [28]	1058	157	901	59 (24.79%)	98 (11.59%)	2.43 (1.66–3.53)	< 0.001
**10**	Al- Oqaily MA [23]	308	258	50	95 (36.82)	0 (0%)	58.99 (7.35-infinity)	< 0.0001

N and n: Number, CI: Confidence interval; RA+: People with rheumatoid arthritis; RA-: People without rheumatoid arthritis; RA+ & T+: People with rheumatoid arthritis and *Toxoplasm*a positive; RA- & T+: People without rheumatoid arthritis and *Toxoplasm*a positive; OR: Odds ratio; NS: Not significant; S: Significant

Our findings suggest that *T*. *gondii* may trigger a pathologic process in individuals, which can ultimately lead to RA. This finding has been reported in other autoimmune diseases such as diabetes mellitus [34, 35], lupus erythematosus [36], and autoimmune thyroid diseases [37]. The higher prevalence of *T*. *gondii* in people with chronic diseases can be explained by the following reasons: 1) toxoplasmosis can contribute to the progression of chronic diseases and 2) treatment of these diseases with immunosuppressive drugs increases the susceptibility of patients to infections, including toxoplasmosis [6]. Recent treatments for RA patients with anti-tumor necrosis factor-α (TNF-α), which leads to brain toxoplasmosis, are indicative of this issue [27, 38]. On the other hand, some toll-like receptors (TLRs) have been identified in mammals, for which some pathogens act as ligands, and as a result of binding between the TLRs and pathogens different types of immune responses can be induced. Based on reference, *T*. *gondii* may be used as ligands for TLRs, which can induce inflammatory response [39].

Also, studies show that *T*. *gondii* increases the expression of interleukin 17 (IL-17) in patients [40], and since this cytokine is involved in the pathogenesis of many autoimmune diseases, including RA [41], a significant relationship between toxoplasmosis and RA can be explained.

RA patients have autoantibodies and rheumatoid factors in their blood [42]. In two studies, these disease activity markers were found to have a significant relationship with toxoplasmosis, especially in high titers [24, 25]. This indicates that *T*. *gondii* can induce or exacerbate arthritis symptoms [43–45].

Because in the studied articles the relationship of age and sex with the prevalence of toxoplasmosis was not evaluated, we avoided the meta-analysis of these risk factors. In addition, diversity in the quality of studies and methods of measuring antibodies limited the interpretation and analysis of these items. These two issues were the important limitations of our meta-analysis.

Despite the significant relationship found between *T*. *gondii* infection and RA in this systematic review and meta-analysis study, further studies are needed on the following grounds: 1) the limited sample sizes in the articles, 2) difference in the quality of the reports, 3) diverse methods of measuring anti-parasitic antibodies, and 4) lack of evaluation of various risk factors such as age and gender.

### Conclusions

One of the most important achievements of our study is that although *T*. *gondii* infection affects about one-third of the world’s population and possibly causes and exacerbates the symptoms of RA, only few studies have addressed this subject. These studies were conducted only in Latin America, Europe, and few regions of Asia and Africa. Accordingly, further studies are needed to achieve accurate results from other parts of the world. Also, further studies will be necessary to clarify the pathogenesis of *T*. *gondii* in humans to understand whether *T*. *gondii* is a cofactor in the development of autoimmune diseases.

## Supporting information

S1 Checklist STROBE statement-checklist. (DOCX)

S2 Checklist PRISMA 2009 checklist. (DOC)
